# Characterization of the glutathione S-transferase gene family through ESTs and expression analyses within common and pigmented cultivars of *Citrus sinensis* (L.) Osbeck

**DOI:** 10.1186/1471-2229-14-39

**Published:** 2014-02-03

**Authors:** Concetta Licciardello, Nunzio D’Agostino, Alessandra Traini, Giuseppe Reforgiato Recupero, Luigi Frusciante, Maria Luisa Chiusano

**Affiliations:** 1 Consiglio per la Ricerca e la sperimentazione in Agricoltura - Centro di ricerca per l'Agrumicoltura e le Colture Mediterranee (CRA-ACM), Corso Savoia 190, 95024 Acireale, Catania, Italy; 2 Consiglio per la Ricerca e la sperimentazione in Agricoltura - Centro di ricerca per l'Orticoltura (CRA-ORT), via Cavalleggeri 25, 84098 Pontecagnano, Salerno, Italy; 3 Dipartimento di Agraria, Università degli Studi di Napoli “Federico II”, Via Università, 100, 80055 Portici, Naples, Italy; 4 Wellcome Trust Genome Campus, Hinxton, Cambridgeshire CB10 1SA, UK

**Keywords:** Sweet orange, GST, Expressed sequence tag, Gene family, Anthocyanin vacuolarization

## Abstract

**Background:**

Glutathione S-transferases (GSTs) represent a ubiquitous gene family encoding detoxification enzymes able to recognize reactive electrophilic xenobiotic molecules as well as compounds of endogenous origin. Anthocyanin pigments require GSTs for their transport into the vacuole since their cytoplasmic retention is toxic to the cell. Anthocyanin accumulation in *Citrus sinensis* (L.) Osbeck fruit flesh determines different phenotypes affecting the typical pigmentation of Sicilian blood oranges. In this paper we describe: i) the characterization of the GST gene family in *C. sinensis* through a systematic EST analysis; ii) the validation of the EST assembly by exploiting the genome sequences of *C. sinensis* and *C. clementina* and their genome annotations; iii) GST gene expression profiling in six tissues/organs and in two different sweet orange cultivars, Cadenera (common) and Moro (pigmented).

**Results:**

We identified 61 GST transcripts, described the full- or partial-length nature of the sequences and assigned to each sequence the GST class membership exploiting a comparative approach and the classification scheme proposed for plant species*.* A total of 23 full-length sequences were defined. Fifty-four of the 61 transcripts were successfully aligned to the *C. sinensis* and *C. clementina* genomes. Tissue specific expression profiling demonstrated that the expression of some GST transcripts was 'tissue-affected' and cultivar specific. A comparative analysis of *C. sinensis* GSTs with those from other plant species was also considered. Data from the current analysis are accessible at http://biosrv.cab.unina.it/citrusGST/, with the aim to provide a reference resource for *C. sinensis* GSTs.

**Conclusions:**

This study aimed at the characterization of the GST gene family in *C. sinensis*. Based on expression patterns from two different cultivars and on sequence-comparative analyses, we also highlighted that two sequences, a Phi class GST and a Mapeg class GST, could be involved in the conjugation of anthocyanin pigments and in their transport into the vacuole, specifically in fruit flesh of the pigmented cultivar.

## Background

Glutathione S-transferases (GSTs) represent an ancient and ubiquitous gene family encoding ~ 25 to 29 kDa proteins that form both homodimers and heterodimers *in vivo*[1]. GST enzymes were first discovered in animals, in the 1960s, for their importance in the metabolism and detoxification of drugs [2]. Their presence in plants was recognized shortly afterwards, when a GST activity was shown to be responsible for protecting maize from injury by the chloro-S-triazine atrazine herbicide [3]. Thereby, GSTs were considered as detoxification enzymes, which are liable for the inactivation of toxic chemical compounds by catalysing their conjugation to GSH. This process results in less toxic and more water-soluble molecules, which are then recognized and transferred across membranes for excretion or sequestration by ATP-dependent membrane pumps. In animals, this mechanism allows the molecules excretion from the body, while in plants, glutathione S-conjugates are sequestered in the vacuole or transferred to the apoplast (storage excretion) [4].

GSTs recognize not only reactive electrophilic xenobiotic molecules (i.e. drugs or herbicides) but also compounds that are of endogenous origin. As an example, in plants, many secondary metabolites are phytotoxic, even to the cells that produce them, and thereby their targeting to the vacuole is crucial [5]. Anthocyanins require GSH conjugation for their transport into the vacuole since their cytoplasmic retention is toxic to the cell and prevents the synthesis of new anthocyanins. This was demonstrated first in 1995 by [6] who suggested that maize gene *bronze2* (*bz2*) was a GST involved in vacuolar transfer of anthocyanins. Furthermore, Mueller et al. [7] evidenced that the GST *Anthocyanin9* (*AN9*) from *Petunia hybrida* is a flavonoid-binding protein that was required for an efficient anthocyanin exportation from the site of synthesis (i.e. cytoplasm) in the vacuole where it is permanently stored.

Because GSTs are remarkably versatile in the recognition of substrates [6], the cloning and the sequencing of these enzymes were undertaken in many plants. McGonigle et al. [8] performed a systematic and comprehensive analysis of the GST multi-gene family in soybean and maize. All the sequences identified by a genomic approach were classified into four categories: type I, II, III and IV according to the criteria in [9] and [10], which are based on amino acid sequence identity and conservation of gene structure (i.e. exon/intron numbers). This classification scheme has been under-way amended and refined into seven classes, six of which include soluble (cytoplasmic) proteins and one microsomal proteins [11,12].

Soluble enzymes are grouped into Tau, Phi, Zeta, Theta, Lambda and DHAR classes.

Phi and Tau are plant-specific classes representing Type I and Type III GSTs, respectively. They are the most representative classes in terms of number of sequences. They conjugate a diverse array of xenobiotics and influence the effects of herbicides on crops and weeds. These GSTs are functionally similar to the drug-metabolizing GSTs found in animals [12]. They also participate in endogenous cellular metabolism [13] by functioning as glutathione peroxidases (GPOXs) that counteract oxidative stress, as flavonoid-binding proteins [7], as stress-signalling proteins [14], and in regulating apoptosis [15].

Zeta class indicates Type II GSTs, while Type IV class [16] was termed Theta, for its strong similarity to the mammalian Theta class. Zeta class GSTs are involved in tyrosine degradation, catalysing the GSH-dependent conversion of malelyacetoacetate to fumarylacetoacetate and thus acting as GSH-dependent isomerases.

Dixon et al. [13], comparing the human Omega GSTs to the Arabidopsis genome, identified two additional plant GST classes: Lambda and DHAR (firstly characterized in rice by [17]).

All the classes mentioned belong to the soluble or cytosolic GST subfamily. A less numerous subfamily is represented by the microsomal GSTs, belonging to the Mapeg class [18], which exhibits transferase and peroxidase activities.

In the present paper we characterized the *Citrus sinensis* (L.) Osbeck GST gene family by screening a collection of 94,127 expressed sequence tags (ESTs). Thanks to the availability of the draft genome sequences of both *C. sinensis* (http://www.citrusgenomedb.org/node/1) and *C. clementina* (http://www.phytozome.net), the transcripts we identified by an EST-based analysis were also aligned to the genome sequences and compared to the currently available genome annotations. GST class assignments were performed and SemiQuantitative (SemiQ) Reverse Trascription (RT) – Polymerase Chain Reaction (PCR) analyses were carried out to assess the expression of GST encoding transcripts in the albedo, flavedo, flesh, young and adult leaves and ovary. Moreover, the transcriptional levels were compared in two different cultivars of sweet orange, Cadenera (common) and a nucellar selection of Moro (pigmented). Pigmented sweet oranges represent a distinctive characteristic of Sicilian accessions. Red pigmentation is typical of fruits from Moro, Sanguinelli and Tarocco cultivars, and the tissues that are highly pigmented are flesh and flavedo. However, a pigmented fruit flesh not always corresponds to an equally pigmented flavedo. Since GSTs may have implications on the pigmentation of the different tissues, disclosing their expression patterns is an important prerequisite for further clarification of the functionality of the anthocyanin biosynthetic pathway in the two cultivars.

## Results and discussion

### *In silico* identification and characterization of *C. sinensis* GST transcripts

The number of GSTs and the multiplicity of their functions in plant genomes are surprisingly large [13]. The question of whether they are all expressed can be addressed by querying EST databases [19] and/or by experimental analyses [8,20]. In the present work we used both computational and experimental analyses to identify, classify and validate tissue expression profiling of *C. sinensis* GST encoding sequences. The whole procedure is traced in the Additional file 1.

Different members of the GST gene family were identified by screening a collection of 94,127 EST sequences from *C. sinensis*. 370 ESTs, putatively encoding GST proteins, were filtered out and used to feed a clustering/assembling procedure to attempt the definition of full-length transcripts. Sixty-two putative unique transcripts, including 28 ESTs assembled into tentative consensus sequences (TCs), and 34 single ESTs (sESTs), were defined. The Tau and the Phi classes show the highest number of unique transcripts: 29 and 20, respectively, confirming evidence from other plants [19,21]. The remaining classes, Lambda (6 sequences), Zeta (3 sequences), Theta (2 sequences) and Mapeg (2 sequences), have fewer components.

The collection of the 62 transcripts was compared versus the GenBank non-redundant nucleotide database in order to identify sequence data, to be exploited to further elongate the 62 transcripts. This analysis also highlighted that TCs CITSI00:1 and CITSI02:1 were contiguous sequences, that can be collapsed into a unique transcript corresponding to the sequence DQ198153 from GenBank. As a consequence, the total number of unique transcripts was reduced to 61 sequences. It is interesting to note that the transcript DQ198153 is exclusively represented by ESTs from fruit flesh. To be precise, these ESTs come from the SSH library described in [22] and appear to be Moro specific.

All the transcripts in each GST class were multiple aligned and analysed so to define the mRNA structure in terms of 5′ UTR, coding region and 3’ UTR segments. This task was also supported by the detection of the longest open reading frame (ORF).

**Figure 1:**
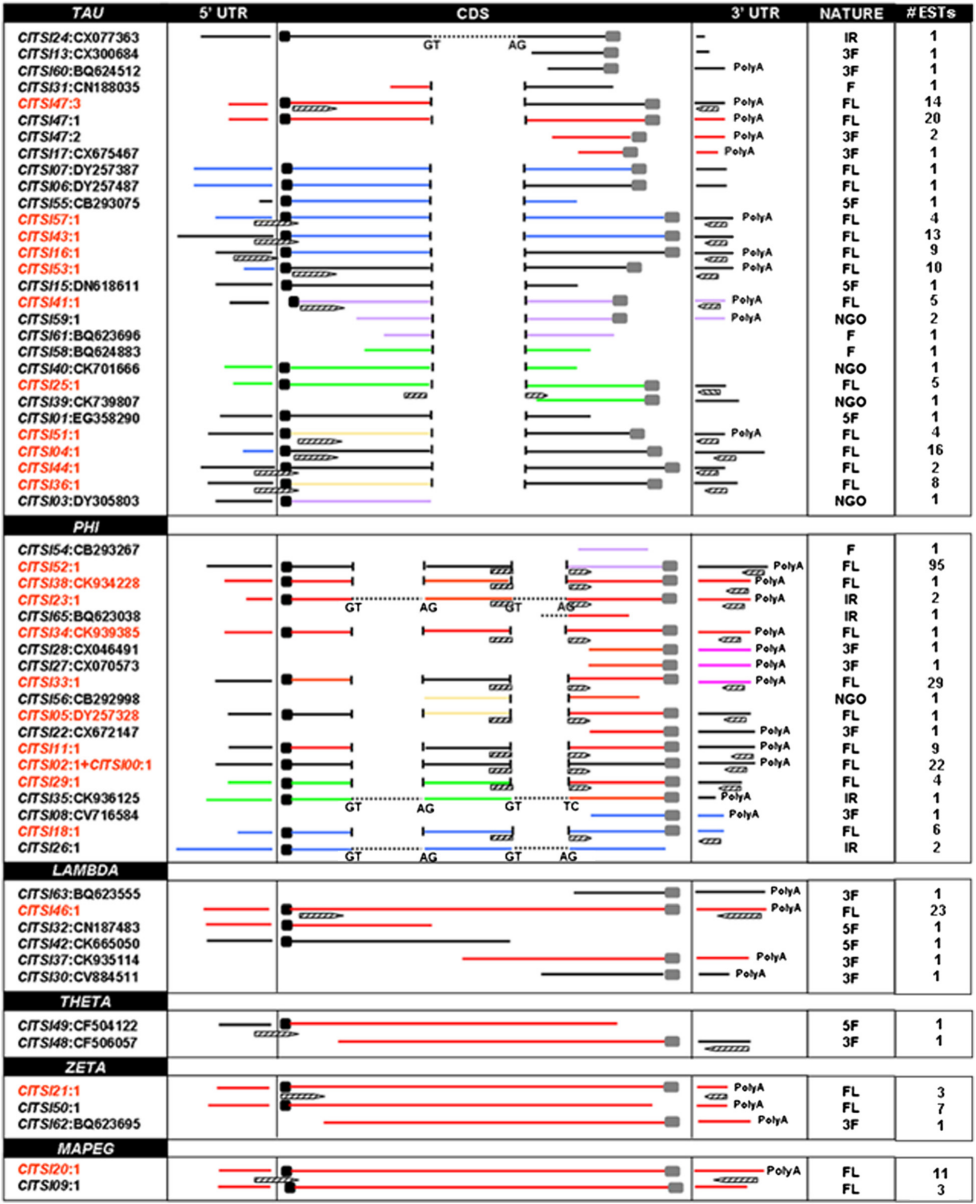
**Schematic view of the six independent multiple alignments generated by grouping the 61 transcripts according to GST class membership.** The coding exons, the 5′ and 3′ UTRs (mRNA segments) are reported. Closely related segments (defined using a cut off of 15% divergence) are reported with the same colour within each class. In red we marked the IDs of those sequences that were analysed by SemiQ RT-PCR. Primers (zebra bars) and their localization along the corresponding GST transcript are shown. The ‘NATURE’ column describes each GST sequence as *full length* (FL), *5′ fragment* (5 F), *3′ fragment* (3 F), *fragment* (F), *no good ORF* (NGO), *intron retaining* (IR), while the last column describes the number of EST per each transcript.

The six different multiple alignments, generated by grouping the 61 transcripts according to the GST class membership, can be scrolled at http://biosrv.cab.unina.it/citrusGST/, while a schematic view summarizing the structures and the similarity relationships among the segments within each class is reported in Figure 1. Each mRNA sequence is represented by a bar that is split into segments corresponding to protein coding exons, 5′ and 3′ UTRs. In addition, each transcript is associated to a tag describing the full- or the partial-length nature of the sequence as inferred from the ORF analysis. The transcripts defined as *full-length* mRNAs (FL, Figure 1) are the ones showing a complete ORF. The remaining transcripts exhibited partial ORFs. Those including the ATG starting triplet but lacking the stop codon were classified as *5′ fragments* (5 F). On the other hand, those transcripts lacking the initiating ATG, but presenting a termination triplet, were classified as *3′ fragments* (3 F). The remaining segments were classified as *fragment* (F). Finally, transcripts showing interspersed stop codons were classified as *no good ORFs* (NGO).

Pairwise DNA distances were calculated and highly similar sequence segments were identified using a cut-off of 15% divergence. In Figure 1, highly similar sequence segments within each GST class are reported with the same colour. However, the sequence similarity obtained by this analysis was not sufficient to collapse these sequences into a unique transcript during the EST assembly step. Sequence variability is further highlighted by the nucleotide sequence alignment available at http://biosrv.cab.unina.it/citrusGST/.

Multiple alignments revealed that one class Tau GST (CITSI24:CX077363) and three class Phi GSTs (CITSI23:1; CITSI35:CK936125; CITSI26:1) could be intron-retaining (IR) sequences. This is also confirmed by the ORF analysis and by BLAST searches that showed the absence of similarity between the putative intron regions and peptides available in protein databases. Putative introns (dashed lines) as well as splice acceptor/donor sites, were indicated in Figure 1. The intron retained by CITSI24:CX077363 is limited by the canonical GT-AG sites. Within the Phi class, CITSI23:1 is an intron retaining isoform compared to CITSI38:CK934228; it was generated by assembling two ESTs (CX671312 and CX671311) and retains both the intron 1 and 2 being the first intron supported by CX671312 and the second by CX671312 and CX671311. The sequence CITSI34:CK939385 is highly similar to both CITSI38:CK934228 and CITSI23:1 sequences (first and second intron excluded), even if several polymorphisms in the 5′ UTR, CDS and 3′ UTR are evident. The singleton CITSI35:CK936125 is considered the intron retaining variant of CITSI29:1, though it is characterized by a non-canonical acceptor site (TC) in the second intron. CITSI26:1, built by assembling 6 ESTs, is the intron retaining isoform of CITSI18:1.

### Genome based assessment of the citrus GST collection

The list of the 61 GST-encoding transcripts and a larger subset of ESTs putatively encoding GSTs (1,611 from a total of 213,830 EST sequences), derived from an independent update of the EST collection (see Methods), were aligned along the *C. sinensis* genome.

This allowed to compare sweet orange gene models (see Methods) and two custom genome annotation tracks, representing the 61 GST encoding transcripts we assembled, and 1,611 ESTs putatively encoding GSTs on the basis of their alignment to genomic sequences. All these annotation tracks can be accessed through the web based resource we made available.

The current *C. sinensis* genome annotation consists of 62 GST gene *loci* with a total of 84 possible transcripts. Twenty-eight of the GST genes have correspondence to one or more transcripts from our collection; 13 are gene *loci* whose exon/intron structure is validated by EST evidence only; 21 are GST gene *loci* with no EST evidence, 7 of which resulted to have a gene structure defined only by one exon.

Furthermore, some of the transcripts we described are associated to the same gene *loci* and it is interesting to note that this is valid also for full-length transcripts. This means that, although the transcripts present differences at sequence level, many of them can be associated to a single *locus* revealing the high level of heterozygosity of the *C. sinensis* genome and/or the high frequency of alternative transcripts in the orange genome. A short class-by-class description of the results follows (see also Additional file 2).

### Tau class

Twenty-one out of the 29 transcripts from the Tau class were aligned versus 14 gene *loci* from *C. sinensis*. Moreover, among the remaining 8 transcripts, 5 were mapped on the *C. clementina* genome.

Specifically, the pairs of sequences CITSI16:1 and CITSI55:CB293075, CITSI25:1 and CITSI40:CK701666, CITSI58:BQ624883 and CITSI39:CK739807 and CITSI47:1 and CITSI31:CN188035 are transcribed from the same *locus* and their exon/intron structure overlaps the one of the corresponding gene. For this reason and considering results from the DNA distance matrix, it is very likely that these transcripts represent allelic isoforms of the corresponding *locus*.

CITSI53:1, CITSI36:1 and CITSI01:EG358290 reflect the exon/intron structure of the corresponding predicted genes.

A cluster of three Tau class GSTs (CITSI41:1; CITSI59:1; CITSI61:BQ623696) was successfully aligned in the same genomic region. In the region, two different gene structures were also predicted.

Sequences CITSI51:1 and CITSI04:1 correspond to the genes *orange1.1g036834m* and *orange1.1g027333m,* respectively.

The singletons CITSI60:BQ624512 and CITSI13:CX300684 were aligned in the same genomic region where a partial gene (only one exon) is predicted. Interestingly, both of the sequences are longer than the predicted gene.

CITSI61:BQ623696 matches the *C. sinensis* genome in three different scaffolds, finding similarity with GST annotated genes. CITSI13:CX300684 overlaps the second exon of the corresponding predicted gene, while CITSI15:DN618611 corresponds to the first exon of the predicted gene in the same *locus*.

CITSI47:2 and CITSI17:CX675467 were aligned in the same genomic region and have correspondence to the second exon of the predicted gene. Finally, no alignments were obtained versus *Citrus sinensis* for the remaining eight sequences from our collection.

To this end, we decided to exploit the *C. clementina* genome in order to check the existence of genes that could be related to these transcripts. Five transcripts out of 8 were aligned along this genome overlapping *glutathione S-transferase* annotated *loci*, revealing the still incomplete nature of the *C. sinensis* genome.

### Phi class

All transcripts we defined from the Phi class were aligned to the *C. sinensis* genome except CITSI11:1, which was successfully mapped on the *C. clementina* genome.

CITSI52:1 and the very short singleton CITSI54:CB293267 are transcribed from the same *locus*. A cluster of eight Phi class GSTs was successfully aligned in the same genomic region, where two different gene models are available.

CITSI02:1 presents the same exon/intron structure of the gene *orange1.1g041226m*. Sequences CITSI29:1 and CITSI35:CK936125 are associated to the same *locus*. Two gene structures were predicted in the same region: the first one includes three exons and reflects the structure of CITSI29:1; the second retains intron II as CITSI35:CK936125 does. The spliced alignment of CITSI18:1 determines three exons, exactly as in the case of the predicted gene. However, two additional sequences CITSI26:1 and CITSI08:CV716584 were aligned in the same region. Both of them describe a unique exon.

CITSI22:CX672147 corresponds to the third exon of the predicted gene in the same *locus*.

### Lambda class

CITSI42:CK665050 was successfully aligned in a region that includes two predicted gene structures. CITSI37:CK935114 and CITSI63:BQ623555 gene structures, even though partial, reflect the ones of the corresponding predicted genes.

The spliced alignment of CITSI30:CV884511 along the reference genome determines four exons, one more than the predicted gene. Finally, in case of sequence CITSI46:1 and CITSI32:CN187483 no spliced alignment was obtained.

### Theta class

No spliced alignment was obtained for both singletons from the Theta class GSTs.

### Zeta class

All the three Zeta class GSTs are transcribed from the same *locus*. In this region ten different gene structures were predicted.

### Mapeg class

Both the Mapeg sequences are associated to the same *locus* and their exon/intron structure duplicates the one of the corresponding predicted gene. It is very likely that they represent allelic variants since they could not be assembled into one single consensus sequence, during the EST processing.

### Expression profiling of full-length transcripts in different tissues/organs in Cadenera and Moro cultivars

In order to address the expression profiling in citrus, we performed SemiQ RT-PCR analyses, considering six different tissues/organs (albedo, flavedo, flesh, young leaf, adult leaf and ovary). Similar experimental approaches are reported for model plant species (Arabidopsis and rice) and further important crops, such as maize and soybean [8], in which GST expression patterns were analysed in a variety of tissues, developmental stages and environmental conditions [21,23]. Moreover GST family-based expression analyses were performed in Arabidopsis [24], poplar [25] and rice [23], while transcript profiling of all the family members are not available in other plants [26].

Here we considered expression in two different cultivars of sweet orange: Cadenera and Moro, common and pigmented ones, respectively. The analysis aimed to point out differences with respect to the different tissues/organs as well as to the two cultivars, especially because GSTs may have implication in differential anthocyanin accumulation. Recently, Butelli et al. [27] demonstrated that blood orange phenotype results from the insertion of an LTR retrotransposon in the promoter region of the *Ruby* gene (which encodes for a MYB transcription factor) and that this gene is specifically activated in fruit in a cold-dependent manner. The implication of cold temperatures in activating the anthocyanin pathway was recently discussed also in [28]. However, it is very likely that *Ruby* itself is necessary, but not sufficient, in the determination of the anthocyanin pigmentation of the flesh fruit and that GST proteins may play a crucial role in the definition of the phenotype. Indeed, Sun et al. [29] proved that the Arabidopsis GST encoding Transparent Testa 19 gene *(TT19)* acts as a carrier to transport anthocyanins to the tonoplast. In addition, *TT19* mutations do not modify the expression level of genes implicated in anthocyanin biosynthesis but at the same time limit anthocyanin accumulation since they cause the transformation of anthocyanin precursors into flavonols.

Among those sequences that resulted to be full-length mRNAs, the ones whose identifier in the Figure 1 is highlighted in red were considered for analyses based on SemiQ RT-PCR experiments. Because of the lack of full-length sequences, no SemiQ RT-PCR experiments were carried out for sequences included in the Theta class.

In Figure 1, primers are drawn as zebra bars below the line representing the sequence to be amplified. Forward primers within the Phi class were selected over the second and the third coding exons, to detect the expression of the mature transcripts. Forward primers within the Tau class were selected over the 5′ UTR and the ATG initial codon, because of higher sequence variability in the 5’ UTR regions that could ensure a higher specificity in primer selection. In Table 1 primer characteristics are indicated.

**Table 1:** List of sequences and primers designed to perform SemiQ RT-PCR experiments

**GST class**	**Sequence ID**	**Primer features**			
		**Forward (5′-3′)**	**Reverse (5′-3′)**	**Annealing temperature (°C)**	**Expected amplicon (nt)**
Tau	CITSI24:CX077363	TCGGCATGGAAGAAGTGAAGCTACTGGGA	ACCGTTCAAAAGATAGTGTCTATGAAGTA	65	784
	CITSI47:3	TCGCCAAGCCCATTTGTGATGAGGGCA	ACGAGACAGGCTGCTGCCTAGTCCGA	66	866
	CITSI57:1	AGTAAGCTTCTGTAATAATGGCGGACGA	ACAATACCCTAAGATAACAGTCGGGGACA	64	879
	CITSI43:1	TCTGTCACAATGGCGGACGAAGTGGT	AGCAGGCAGCACGATTGCGCTGCT	68	732
	CITSI16:1	TCACTCGCCCTTAATTCTCAGTAAGGT	AGATTGACGCCACATAATATTCCCA	60	966
	CITSI53:1	TGCTGGGTTACTGGGCAAGCCCCT	ACCTTCATGCATGGGCAACCGCTGA	66	686
	CITSI41:1	GTTTACAGGGTGATTTGGGCTCTGA	ACCACTATGCTAGTCCCCCGAACT	63	757
	CITSI25:1	AAGCTCTATGGCACATGGGTAAGCCCTT	ACACAGAGAGAGAGCTAACCCAATCA	65	730
	CITSI51:1	TGGCCAAGCCCGTTTGTGTTTAGGGT	AGACTTTCCACACAACATCACACTAC	63	756
	CITSI36:1	GCGAAAATAATATGGCCAAAGAAGTGACGCT	GTCATTACAACACACCACAACACCACCT	64	765
	CITSI04:1	AGACGTGGTCAAGCCCCTTTGGT	TGGAATGGGAAAAGGGCAAAAGGA	62	889
	CITSI44:1	GCAGAAGATTATGGCAACAAAAGTG	GAGCGTACAGAAAGGAGACACGTGCA	62	764
Phi	CITSI52:1	AAGCTCTTCGAATCAAGGGCAATCACCCAA	TTTTGATAAACCCATTGGGACAGTCGT	65	800
	CITSI38:CK934228	ATACGAATCTCGAGCTATCATAAGGTACTA (^a^)	TTTTCTCCCAAGGCCCCAAGCATTT	64	636
	CITSI23:1	ATACGAATCTCGAGCTATCATAAGGTACTA (^a^)	TTTTGTCCTCCAAGGCCCCAAGCAT	64	638
	CITSI34:CK939385	ATACGAATCTCGAGCTATCATAAGGTACTA (^a^)	TTTTGTCCTCCAAGGCCCCCAGCAT	65	678
	CITSI33:1	TTTATACGAGTCGCGAGCTATCATGAGGT	TTTTAAAGCTCCAACTCCAAACAT	60	658
	CITSI05:DY257328	ATTTTATACGAGTCGCGAGCTATCATGAGG	ACCCCTTATCCAAGGAACATTTCCCA	64	565
	CITSI11:1	TGGGGATTTTACTCTATACGAATCGCGA	ATGGCGACAACAAGAAATCGCCGCA	63	640
	DQ198153 (CITSI02:1 + CITSI00:1)	GCTTTTTGAGTCGAGGGCAATCATAAGG	GATAACAGTAATGACAGCCAGCCGAA	64	549
	CITSI29:1	AGGATGAGAAAATCTCCCTCTTAGAGTCT	TGGGAAATTATTAGACCATGCCA	63	748
	CITSI18:1	ATTTGAATCTAGAGCAATGACAGCATACGT	CATCAATGTAAAATCATCACGCAACCA	63	599
Lambda	CITSI46:1	CCTCCAAGATAGGCCCGCTTGGTAC	TCCAGCAACGTACACAAGCTCACATCGGCA	66	672
Zeta	CITSI21:1	ATGCTGAAACTGTATTCATACTGGAGGAGT	TGCTGCTTATTGAGGGTCAACAAAGGCTG	64	880
Mapeg	CITSI20:1	CGACTCGACTATGGCGGATGCAAC	CTATGAGCTTATGCTTGCGCCATGCAGC	66	638

GST classes, oligonucleotides sequences and the expected size of amplicons (on the basis of correct maturation) are highlighted. Uppercase letters indicated the use of the same forward primer.

Expression patterns for each of the 25 citrus GST transcripts are summarized in Table 2. Electrophoresis gel images from the experimental analysis are reported at http://biosrv.cab.unina.it/citrusGST/. Positive symbols showed the expression at different cycles ['+' (weak) to '++++' (strong)] depending on the amplicon presence at the PCR cycles 20, 25, 30 and 35. Negative symbol indicates absent expression.

**Table 2:** Summary of tissue/organ expression profiling by SemiQ RT-PCR

								
**GST Class**	**Sequence ID**	**Cultivar**	**SemiQ RT-PCR**					
			**Albedo**	**Flavedo**	**Flesh**	**Young leaf**	**Adult leaf**	**Ovary**
Tau	CITSI24:CX077363	C	+	+	++	+++^a^	++++^a^*	++++^a^*
		M	+	+	+++	++^a^	++^a^*	++^a^*
	CITSI47:3	C	++	++	++	++^a^	++	+++^b^*
		M	+^a^	++	+	++^a^	++	-*
	CITSI57:1	C	++^a^	+^a^	+++^a^	++^a^	++++^a^	++++^a^*
		M	++^a^	++^a^	++++^a^	++^a^	+++^a^	-*
	CITSI43:1	C	++	++	+++	+++	+++	++++^a^*
		M	++	++	+++	+++	+++	++*
	CITSI16:1	C	+++	-	+++	+++	+++	-
		M	+++	-	++	+++	+++	-
	CITSI53:1	C	+++	+++	+++	+++	+++	+++^a^
		M	+++	+++	+++	+++	+++	+++
	CITSI41:1	C	+	++	+++	++^a^	+++^a^	+++^a^
		M	+	++	+++	++^a^	++	++
	CITSI25:1	C	++	+	+	+++	++	+
		M	+	+	++	+++	++	++
	CITSI51:1	C	++	++	++	-	++	++^b^*
		M	+	++	++	-	++	-*
	CITSI04:1	C	-	-	-	-	++	-
		M	-	-	-	-	+	-
	CITSI44:1	C	++	++	+++	++	+++	++^b^*
		M	+++	+++	++	+++	+++	-*
	CITSI36:1	C	++	++	+++	++++	+++	+++^a^
		M	+++	++	+++	++++	+++	++
Phi	CITSI52:1	C	+	+	+	++	++	+
		M	-	+	+	++	++	+
	CITSI38:CK934228	C	-	+	++*	++	+++	+
		M	-	+	-*	++	++	-
	CITSI23:1	C	-	+	++	++	++	+
		M	-	+	+	++	++	-
	CITSI34:CK939385	C	-	+	-	++	+++*	+
		M	-	+	-	+	+*	-
	CITSI33:1	C	+++	++	+++	+++	++	+++
		M	+++	++	++	+++	+++	++
	CITSI05:DY257328	C	++	++	++	++	++^a^	++
		M	++	++	++	++	++	+
	CITSI11:1	C	+	-	++	-	+	++
		M	+	+	++	+	+	+
	DQ198153	C	-	-	+*	+	+	-
		M	-	+	++++*	+	+	-
	CITSI29:1	C	-	-	+	++	+++	+
		M	-	-	+	++	++	+
	CITSI18:1	C	-	-	-	-	+	+
		M	-	-	-	-	-	-
Lambda	CITSI46:1	C	+++	+++	++++	++	++++	++++
		M	+++	+++	++++	+++	++++	+++
Zeta	CITSI21:1	C	++^a^	+^a^	+++^a^	++^a^	++^a^	++^a^
		M	++^a^	++^a^	+++^a^	++^a^	++^a^	++^a^
Mapeg	CITSI20:1	C	++	++	+*	++	+++	+++
		M	+++	++	+++*	+++	+++	++

‘C’ and ‘M’ indicate the Cadenera and Moro cultivars, respectively. Expression level is indicated as '–' (no expression) or from '+' (weak) to '++++' (strong) depending on the presence of the amplicon at the PCR cycle 20 (+), 25 (++), 30 (+++) and 35 (++++).

Uppercase letters indicate:

^a^the presence of multiple-bands.

^b^RT-PCR products with size greater than the expected on the basis of the primer design on the transcripts.

*differences in the expression levels of more than ten cycles between the two cultivars.

The results presented in Table 2 show two different aspects: transcripts presence/abundance (*i*) in various tissues/organs and (*ii*) in both cultivars. In the case of Tau class GSTs, the expression is detected in all the tissues/organs, with the only exception of CITSI04:1, which results expressed only in adult leaves of both cultivars; CITSI51:1, which is not expressed in young leaves of Cadenera and Moro, and in ovary of common orange; CITSI16:1, which is not present in flavedo and ovary of both cultivars. Considering the cultivar related expression, differences of more than ten cycles are indicated by asterisks in Table 2. Moreover, some of the transcripts were not detected at all in the ovary samples from Moro, such as CITSI47:3, CITSI57:1, CITSI51:1 and CITSI44:1 (indicated by ‘-’ in correspondence of the cultivar were the expression was not detected in Table 2). Multiple-bands indicated by uppercase a in Table 2) generally occur only in specific organs in both cultivars as in CITSI24:CX077363, CITSI47:3, CITSI57:1 and CITSI41:1. Expression levels marked by uppercase b (CITSI47:3, CITSI51:1 and CITSI44:1) indicate RT-PCR products in the column 'ovary' with size greater than the expected on the basis of the primer design and the transcripts. Interestingly, in some samples, this behaviour is cultivar specific, as in the ovary of CITSI43:1, CITSI53:1, CITSI41:1 and CITSI36:1 in Cadenera and CITSI47:3 in the albedo in Moro. The presence of multiple bands is not suspicious since, as discussed in the previous section, alternative transcription for citrus GST genes seems to be very common.

Phi class GSTs show more specific expression patterns. Indeed, CITSI33:1 and CITSI05:DY257328 are expressed in all the tissues/organs. CITSI38:CK934228 and CITSI23:1 were detected with similar expression patterns in flavedo and leaves; both transcripts are absent in the albedo of both cultivars and in the ovary of Moro. The only difference in the expression pattern of the two transcripts is the absence of CITSI38:CK934228 in the flesh of Moro. CITSI34:CK939385 follows a similar behaviour with a higher expression in the adult leaf of Cadenera and absence of expression in albedo and in the flesh for both cultivars. Heterogeneous patterns were detected for the remaining transcripts considered in the different tissues/organs, indicating a higher variability in the expression pattern within the Phi class. Table 2 shows remarkable differences (i.e. transcripts with expression differing of more than 10 cycles) between the two cultivars. Higher expression levels were detected in Cadenera for CITSI34:CK939385 in adult leaves, and in the flesh column for the sequence DQ198153 (Table 2). Interestingly, the early over-expression of DQ198153 transcript in the Moro is corroborated by the expression patterns inferred when considering the EST-based tissue/organ distribution, as reported in the section '*In silico* identification and characterization of *C. sinensis* GST transcripts'. Moreover, the low expression detected in flavedo of the Moro cultivar compared to a completely absent signal in Cadenera may be associated to the presence of some pigmentation also in this part of the fruit, in Moro, even if at a lowest extent. This finding suggests that DQ198153 may be responsible for anthocyanin accumulation in the Moro fruit flesh, similarly to the Mapeg GST CITSI20:1, which also resulted over-expressed in the flesh of the Moro fruit. As pointed out by Conn [30], this microsomial GST can be involved in anthocyanin transport to the vacuole or be associated to their biosynthesis at the cytosolic face of the endoplasmic reticulum (reviewed in [31]).

As stated, no expression in the flesh of Moro was evident for CITSI38:CK934228 (Table 2). Moreover, CITSI18:1 is only detected in the adult leaf and in the ovary of Cadenera thus indicating distinct roles of GSTs members in the different tissues of different cultivars. Multiple bands are not common in the Phi class, probably thanks to more reliable gene structures inferred by the computational analysis and, as a consequence, to a more specific primer design (Figure 1). However, multiple-bands are evident only for CITSI05:DY257328 in the adult leaves of Cadenera.

No relevant difference concerning the expression in all the tissues/organs and between Cadenera and Moro is evident in Lambda, Zeta and Mapeg classes.

Double-bands are also evident in the Zeta class (Table 2). This could be a direct result of alternative transcription of the corresponding gene *locus*, as it is clear from what is described in the section 'Genome based assessment of the citrus GST collection'.

As already shown in the Figure 1, some of the citrus GST sequences share a high similarity at nucleotide level (bars with identical colours). This is generally confirmed at protein level (as said after) as often the same sequences cluster together also sharing minimum distances (Table 3). However, it is also evident that the nucleotide sequences do not fall in the same tentative consensus when assembled (see Methods). Indeed, high similar sequences within a heterozygous species, like *C. sinensis*, may also account for allelic isoforms.

**Table 3:** **Minimum distance matrix of GST proteins between *C. sinensis *** **GSTs and other species**

**N.**	**Citrus ID**	**Minimum distance matrix**				**GST Class**
		**Distance matrix value**	**Plant ID**	**Functional involvement**	**Reference**	
1	Cs|CITSI47.3	0.6838	At|NP_172508.1 chr01	Early responsive to dehydratation	-	Tau
2	Cs|CITSI57	0.1692	Cs|CITSI43	-	-	
3	Cs|CITSI43	0.1692	Cs|CITSI57	-	-	
4	Cs|CITSI16	0.2137	Cs|CITSI43	-	-	
5	Cs|CITSI51	0.809	Cs|CITSI36	-	-	
6	Cs|CITSI36	0.809	Cs|CITSI51	-	-	
7	Cs|CITSI44	0.9142	Gm|AAG34802.1	-	-	
8	Cs|CITSI24	0.9067	Gm|AAG34804.1	Induced by infection	[8]	
9	Cs|CITSI41	0.9092	Cs|CITSI24	-	-	
10	Cs|CITSI04	0.6216	Vv|GSVIVP00022906001	-	-	
11	Cs|CITSI53	1.047	Os|AAK98546.1 chr10	-	-	
12	Cs|CITSI25	1.0679	At|NP_180510.1 chr02	Induced after chemical treatments	[34]	
13	Cs|CITSI52	0.8027	At|NP_171792.1_chr01	Early responsive to dehydration	[33]	Phi
14	Cs|CITSI23	0.0737	Cs|CITSI34	-	-	
		0.0737	Cs|CITSI38	-	-	
15	Cs|CITSI33	0.013	Cs|CITSI05	-	-	
16	Cs|CITSI05	0.013	Cs|CITSI33	-	-	
17	Cs|CITSI34	0.0261	Cs|CITSI38	-	-	
18	Cs|CITSI38	0.0261	Cs|CITSI34	-	-	
19	Cs|CITSI11	0.181	Cs|CITSI38	-	-	
20	Cs|DQ198153	0.925	At|NP_197224.1 chr05	Anthocyanins accumulation	[29,35,36]	
21	Cs|CITSI18	0.5275	Gm|AAG34811.1	-	-	
22	Cs|CITSI29	0.9667	Os|AAG32477.1 chr03	-	-	
23	Cs|CITSI46	0.5253	Vv|XP_002275882	-	-	Lambda
24	Cs|CITSI21	0.5359	Vv|XP_002273077	-	-	Zeta
25	Cs|CITSI20	0.5156	At|NP 176758.1 chr01	-	-	Mapeg

Distance matrix value and the relative plant for each GST class are indicated. Known functional involvement and related references are shown, when available.

Similar sequences here considered often show differences in the expression patterns within a cultivar, confirming their structural difference, and indicating different functional roles. As an example, sequences CITSI38:CK934228, CITSI23:1 and CITSI34:CK939385 show similar expression patterns though the primers are associated to regions showing nucleotide differences among the sequences. In particular, CITSI38:CK934228 and CITSI23:1 show expression differences in the flesh tissue when compared to CITSI34:CK939385 in Cadenera, while CITSI38:CK934228 and CITSI34:CK939385 show differences with CITSI23:1 in Moro. More striking differences are evident within the whole group of sequences, as evident from Table 2.

Considering the Tau class, differences in expression within a cultivar are evident within the group of sequences CITSI57:1, CITSI43:1 and CITSI16:1, which prominently diverge in the 5′ UTR and 3' UTR regions, as well as within the group of sequences CITSI51:1, CITSI44:1, CITSI36:1 and CITSI24:CX077363.

In addition, the interesting aspect is that the sequences mentioned may show different behaviors when considering each cultivar, as already shown for the Phi sequences CITSI38:CK934228, CITSI23:1 and CITSI34:CK939385. This is valid also in the Tau class. As an example, sequence CITSI51:1 show lack of expression in the young leaves in both cultivars when compared to the other similar sequences (CITSI44:1, CITSI36:1, CITSI24:CX077363). However, the expression in the ovary is similar to the one reported for sequence CITSI44:1, where the two cultivars show different behaviors.

### Comparative analysis of citrus GSTs and sequences from other plant species

We compared the protein products of the experimentally analysed full-length sequences in our dataset, with GSTs from *A. thaliana*, *Glycine max*, *Vitis vinifera*, *Oryza sativa* and *Zea mays*. In Additional file 3, the number of sequences per species grouped by GST class is reported. Sequence identifiers as well as chromosome positions are indicated when known. All the considered species have representatives within each GST class with the exception of Lambda, Mapeg, Theta and DHAR. Furthermore, Lambda and DHAR classes are represented only in dicots. This is probably due to the different evolutionary fates in each clade [26] or to limits in the annotation available for each of the species considered here. A manually edited multiple alignment was drawn (available at http://biosrv.cab.unina.it/citrusGST/). The alignment shows groups of sequences clearly associated to different GST classes. Moreover, it reveals high heterogeneity within the most representative classes, Tau and Phi.

Sequences from dicots generally group separated from monocots, confirming their independent expansion [26]. In addition, the alignment reveals both sequences that are grouped by species as well as clusters of similar sequences from different species. Sequences revealing high similarity are not necessarily those that co-localize along a chromosome. This highlights that sequence similarities within GSTs in a plant genome cannot be only explained by recent tandem duplication events, as previously described [21], but can be due to sequence conservation linked to specific functional requirements. A distance matrix was also defined from the multiple alignment per each class. In Table 3, the identifiers of sequences showing the minimum distance with the citrus GSTs are reported. In case available, references concerning known roles of the sequences are included in the table. Distance relationships in which citrus GSTs have minimum distances with sequences from other plant species are reported too (e.g. see sequences number 1, 7, 8, 10, 11 and 12 within the Tau class; 13, 20, 21 and 22 within the Phi class; sequence 23 within the Lambda class; sequences 24 and 25 within the Zeta and the Mapeg classes).

In general, GSTs have been studied as stress related genes [8,32-34]. The role reported in the literature for the Tau Class GST sequences At_NP_172508.1, Gm_AAG34804.1 and At_NP_180510.1, which are the most similar to CITSI47:3, CITSI24:CX077363, CITSI25:1, and the Phi class GST At|NP_171792.1 similar to CITSI52:1, is associated to stress. Though the sequence similarity, we cannot confirm this role for the citrus GSTs since the expression patterns here considered do not include tissues under stress conditions. However, a high similarity level was detected between the sequence DQ198153 from *C. sinensis* and sequence NP_197224 (i.e. *TT19*) from *A. thaliana*, which is reported to be involved in anthocyanins accumulation [29,35,36]. The over expression of DQ198153 in Moro flesh when compared to Cadenera (Table 2), as well as the tissue specificity of the ESTs this sequence is assembled from, highlight that the sequence is differentially expressed in Moro flesh fruit, and suggest that this GST may be specifically involved in the accumulation of anthocyanins in the blood cultivar [37]. In fact, it is known that although the anthocyanin’s biosynthetic enzymes are localized in the cytoplasm [38], stable colouration develops only after the pigment is transferred to the vacuole thank to a GST, as observed in maize [39]. The functional role of DQ198153 is also corroborated (i) by its sequence similarity with the Anthocyanin 9 (An9) from *P. hybrida*[22], and (ii) by GST enzyme assays [40]. AN9, indeed, has been the first plant GST proposed to be a cytoplasmic flavonoid carrier protein [7].

## Conclusions

Characterization of the GST gene family was conducted in different plants, considering their well-known involvement in biotic and abiotic stress [11,41-49]. By contrast, not many information is available for citrus [22,40,50]. In this paper, we present the first characterization of the GST gene family in citrus and provide insights on the role of some GSTs in two different cultivars, Cadenera and Moro, which show different phenotypic traits of the fruit in terms of pigmentation.

The 61 GST transcripts we defined were arranged into the six well-known plant GST classes and are described in a dedicated web resource accessible at http://biosrv.cab.unina.it/citrusGST/.

Forty-eight of the defined transcripts were mapped onto the *C. sinensis* genome, corresponding to 28 GST encoding genes. Most of the GST transcripts co-localize in the same gene *loci* confirming both the existence of alternative transcripts for these genes and the highly heterozygous nature of the *C. sinensis* genome. Further six transcripts were successful aligned only to the draft of the *C. clementina* genome revealing the still incomplete nature of the *C. sinensis* genome.

The expression profiling analysis revealed that some transcripts show tissue/organ- and/or cultivar-specific expression patterns.

In particular, our results suggest a cultivar specific role for the sequence DQ198153. This sequence may be involved in the determination of the fruit flesh phenotype of the pigmented cultivar, proven its specific expression and its high sequence similarity with *TT19* from Arabidopsis and *AN9* from petunia. Furthermore, the over-expression of the Mapeg GST CITSI20:1 in the flesh of Moro also suggests its possible cultivar specific involvement in the binding of anthocyanins in this tissue.

The specific role of GST family members associated to anthocyanin vacuolarization is corroborated by related efforts [22,51]. Moreover, our work highlights that GSTs, mainly studied for their involvement in stress response, may show differential expression in different cultivars, addressing the relevance of deeper investigations to understand factors affecting phenotypic variability from members of the same species.

## Methods

### Plant material

Sweet orange tissues/organs were collected from Cadenera (common) and from a nucellar selection of Moro (pigmented) cultivars harvested in the orchard of CRA-ACM located at Palazzelli (Lentini, SR, Italy). For each cultivar, around twenty fruits from three different plants (necessary for biological replications) were collected at the end of January, when the anthocyanin pigmentation of Moro flesh was clearly evident and the anthocyanin content in the flesh was quantified and compared to its absence in Cadenera [22].

### Identification of ESTs encoding putative GST proteins

Members of the *C. sinensis* GST gene family were identified by screening the EST collection, including 94,127 sequences, retrieved from the dbEST division of the GenBank repository (Release 01-11-2006). A preliminary functional annotation was based on BLASTx comparisons versus the UniProtKB/Swiss-Prot database (Release 01-11-2006). The NCBI BLAST report file was parsed with an in-house Perl script in order to select ESTs that matched a GST protein as best hit. The original data-set was reduced to 370 putative GST encoding sequences. This collection was used to feed the clustering/assembling procedure. PaCE (default parameter) is the EST clustering software [52] that we used in order to cluster the ESTs which putatively tag the same gene. Instead, the assembly software CAP3 [53] (with an overlapping window of 30 nucleotides and a minimum score of 95) was used in order to assemble into TCs the ESTs which were grouped in the same cluster.

A second screening was performed on a larger and updated EST collection, comprising 213,830 sequences, downloaded from dbEST (Release 01-08-2012).

### ORF Finding

The EXPASY Translate tool (http://www.expasy.ch/tools/dna.html) was used to define the longest ORF for each GST putative transcript.

### GST class assignment

*Entrez* query was carried out to retrieve all the *A. thaliana* protein sequences belonging to the GST class Tau (resulting in 29 different sequences), class Phi (20 sequences), class Zeta (3 sequences), class Theta (2 sequences), class Lambda (6 sequences) and class Mapeg (1 sequence). All the protein sequences in each class were analysed by Block Maker [54], a tool for the identification of conserved blocks (i.e. segments corresponding to the most highly conserved regions of proteins) in a set of related sequences. An embedded consensus sequence for each of the GST classes was generated using COBBLER (COnsensus Biasing By Locally Embedding Residues [54]). These COBBLER-embedded sequences were used as a reference to classify the putative *C. sinensis* GST sequences into specific GST classes.

### Generation of multiple alignments

The ClustalW program [55] was used to generate multiple alignments of the nucleotide (mRNA) sequences for each GST class. The alignment editor BioEdit [56] was used to edit multiple alignments in order to define the mRNA structure and identify the mRNA segments (5′ UTR, coding region, 3’ UTR).

Segment-to-segment DNA distances were calculated using the DNADIST program in PHYLIP [57]. The cut-off of 15% divergence was used to define two segments as closely related.

### Transcripts to genome alignments

*C. sinensis* whole genome assembly and annotations (JGI v1.0) were retrieved from the Citrus Genome Database (http://www.citrusgenomedb.org/).

GST encoding transcripts were aligned to the reference *C. sinensis* genome scaffolds using GenomeThreader [58] with 80% minimal nucleotide coverage and 80% sequence identity.

### Total RNA extraction

All tissues/organs were taken and frozen in liquid nitrogen. The TRIzol® LS Reagent (Invitrogen, Scotland UK) was used for extracting total RNA from 3 g of flesh according to the manufacturer’s instructions. Total RNA of flavedo and adult leaves were extracted from 2 g of sample using an adjustment of a standard extraction RNA protocol of Cl-Guanidine thiocyanate [59]. The total RNA from the albedo was extracted using Concert (Invitrogen, Scotland UK). The RNeasy Plant Mini kit (Qiagen) was used for the total RNA extraction from 0.1 g of young leaves and ovaries. All the RNAs were treated with DNase (Promega) for 30 min at 37°C to remove genomic DNA.

The amount and quality of the total RNA were estimated by spectrophotometer readings and by agarose gel electrophoresis (0.8% agarose in 1x TAE). The electrophoresis gels were stained with ethidium bromide.

### Expression profiling by semi-quantitative RT – PCR

SemiQ RT-PCR analyses were performed to assess the expression level of the putative GSTs in the albedo, flavedo, flesh, young and adult leaf and ovary. SuperScript III One-Step RT-PCR with Platinum Taq (end point) (Invitrogen) was used. The amplification of the Elongation Factor (EF) alpha chain (AY498567) RNA was used as control (data not shown). The primers used to amplify the target regions are shown in Table 1 where in a row are reported the sequence, the annealing temperature and the amplicon expected size. To be sure to isolate the specific amplicon, two different kind of blast were conducted: firstly of oligos *versus* the cluster sequences, secondly BlastN non-redundant and dbEST types. For RT-PCR reactions, first-strand cDNA was synthesized from 200 ng of total RNA in a volume of 25 μL containing 1x PCR Reaction mix, 0.2 μM of each target-specific amplification primer, 1U of SuperScript III One-Step RT-PCR with Platinum Taq. Reverse transcription was performed at 50°C for 30 min., followed by PCR amplification: denaturation at 94°C for 6 min followed by 35 cycles of denaturation at 94°C for 30 s, annealing for 30 s, and extension at 72°C for 120 s, with a final extension at 72°C for 7 min, in a GeneAmp PCR system 9700 (Applied Biosystems, Foster City, CA, 94404). Five microliters of each sample were analysed at 20, 25, 30 and 35 cycles. SemiQ RT-PCR experiments were repeated three times to be sure about expression level. The amplified cDNA samples were separated by agarose gel electrophoresis (1.5% agarose in 1x TAE) and stained with ethidium bromide.

### Protein alignment and phylogenetic analysis of GSTs from various plant species

The Arabidopsis GST gene information was filtered out from GFF files retrieved from the NCBI FTP site (ftp://ftp.ncbi.nih.gov/genomes/Arabidopsis_thaliana/).

UniProt accession numbers for all the maize GST proteins that are present in the file “Gramene_Protein_Desc.txt” from the GRAMENE FTP site (ftp://ftp.gramene.org/) were retrieved. Then, by using the UniProt accession numbers all the corresponding protein sequences were filtered out from the file “poaceae_sptreml.fa” available at the GRAMENE FTP site, as well. The sequences amount to 42 proteins and correspond to the dataset available in [8].

Protein sequences from soybean were retrieved from the GenBank repository by using the accession numbers reported in [8] as query terms. Sequence J03197 (M20363, Type III GSTs: GH2/4 Gmhsp26-A or GmGST 1) from soybean was discarded since it was no more annotated as a GST.

All the rice GST proteins were retrieved from the Rice Genome Annotation web site (http://rice.plantbiology.msu.edu/) by searching for ‘glutathione S-transferase’. The additional information concerning the locus name, chromosome location and gene coordinates along the *O. sativa* genome were manually retrieved from the same web site. The total sequences collected are 82. In particular, 46 out of 82 protein sequences correspond to GSTs described in [19], who specifically considered a total of 61 sequences. Sequences indicated as NM_1903851 – OsGSTF11, OJ1006_F06.6 – OsGSTF16, AF379376 – OsGSTU6, OSJNBa0018H01.7 – OsGSTMU37, P0493G01.11 – OsGSTF13 and OSJNBa0033P04.19 – OsGSTU36 were discarded since they represent expired records or pseudogenes [23].

*Vitis* protein sequences were downloaded from Grape Genome Browser (http://www.genoscope.cns.fr/externe/GenomeBrowser/Vitis/entry_ggb.html).

The ClustalW program [55] and the alignment editor BioEdit [56] were used to generate multiple alignments considering the collected plant GSTs. For each GST class, the amino acid distance matrix was calculated and the sequences with minimum distance value with *C. sinensis* GST protein sequences were reported. When available, references addressing functional results for a specific GST protein were also considered.

### Web resources

A dedicated web resource has been developed in order to (i) access the catalogue of the 61 GST-encoding transcripts; (ii) display the alignments of transcripts along the reference genome; (iii) scroll multiple sequence alignments per GST class; (iv) allow inspection of SemiQ RT-PCR panels and (v) investigate protein sequence multiple alignment obtained by combining *C. sinsensis* and *A. thaliana*, *G. max*, *V. vinifera*, *O. sativa* and *Z. mays* GSTs. The web resource is accessible at http://biosrv.cab.unina.it/citrusGST/.

## Authors’ contributions

CL carried out the molecular genetic studies, participated in the sequence alignment and wrote the manuscript, NDA was involved in the analysis of the EST sequences, in the organization and the maintenance of the web resource and contributed to write the manuscript; AT aligned the transcripts to the reference genomes and analysed the resulting data; GRR and LF contributed to the realization of the project; MLC conceived the project, coordinated the different efforts and to wrote the manuscript. All authors read and approved the final manuscript.

## Supplementary Material

Additional file 1: Figure S1 Detailed scheme of the procedure used. Click here for file

Additional file 2: Table S1 Sequence alignments along the reference genomes. The matches along the *C. sinensis* genome are reported in details for each sequence, as well as their presence along the *C. clementina* one. Moreover the ‘Seq. Structure description’ and ‘SemiQ RT-PCR’ revealed in the manuscript, is also reported. Click here for file

Additional file 3: Table S2 Schema of GST proteins divided into each class end in relation to dicotyledon (*A. thaliana, C. sinensis, V. vinifera, G. max*) and monocotyledon (*O. sativa, Z. mays*) affiliation deducted after protein alignment. The number of encoded proteins for each plant species, the ID of the protein and the genome position are showed; the chromosome start-end for each transcript when possible is also indicated. In the Phi class some examples of clusterization analysis are indicated (^a^) genes from different chromosomes, (^b-c^) proximal gene and (^d^) alternative transcripts. Click here for file

## References

[B1] Alfenito MR Souer E Goodman CD Buell R Mol J Koes R Walbot V Functional complementation of anthocyanin sequestration in the vacuole by widely divergent glutathione S-transferase The Plant Cell 1998 13 1135 1149 9668133 10.1105/tpc.10.7.1135 PMC144053

[B2] Wilce MCJ Parker MW Structure and function of glutathione S-transferases Biochem Biophys Acta 1994 1205 1 18 8142473 10.1016/0167-4838(94)90086-8

[B3] Frear DS Swanson HR Biosynthesis of S-(4 ethylamino 6-isopropylamino 2-s-triazino) glutathione; partial purification and properties of a glutathione S-transferase from corn Phytochemistry 1970 9 2123 2132 10.1016/S0031-9422(00)85377-7

[B4] Listowski L Abramovitz M Homma H Niitsu Y Intra-cellular binding and transport of hormones and xenobiotics by glutathione S-transferases Drug Metab Rev 1988 19 305 318 10.3109/03602538808994138 3068032

[B5] Martinoia E Grill E Tommasini R Kreuz K Amrhein N An ATP-dependent glutathione S-conjugate ‘export’ pump in the vacuolar membrane of plants Nature 1993 364 247 249 10.1038/364247a0

[B6] Marrs KA Alfenito MR Lloyd AM Walbot V A glutatione S-transferase involved in vacuolar transfer encoded by the maize gene *Bronze-2* Nature 1995 375 397 400 10.1038/375397a0 7760932

[B7] Mueller LA Goodman CD Silady RA Walbot V AN9, a petunia glutathione S-transferase required for anthocyanin sequestration, is a flavonoid-binding protein Plant Physiol 2000 123 1561 1570 10.1104/pp.123.4.1561 10938372 PMC59113

[B8] McGonigle B Keeler SJ Cindy Lau SM Koeppe MK O’Keefe DP A genomics approach to the comprehensive analysis of the glutathione S-transferase gene family in soybean and maize Plant Physiol 2000 124 1105 1120 10.1104/pp.124.3.1105 11080288 PMC59210

[B9] Droog FNJ Hooykaas PJJ Van der Zaal BJ 2,4-Dichlorophenoxyacetic acid and related chlorinated compounds inhibit two auxin-regulated type III tobacco glutathione S-transferases Plant Physiol 1995 107 1139 1146 12228421 10.1104/pp.107.4.1139 PMC157246

[B10] Droog F Plant glutathione *S*-transferases, a tale of Theta and Tau J Plant Growth Regul 1997 16 95 107 10.1007/PL00006984

[B11] Edwards R Dixon DP Walbot V Plant glutathione transferases: enzymes with multiple functions in sickness and in health Trends Plant Sci 2000 5 193 198 10.1016/S1360-1385(00)01601-0 10785664

[B12] Edwards R Dixon DP Plant glutathione transferases Methods Enzymol 2005 401 169 186 16399386 10.1016/S0076-6879(05)01011-6

[B13] Dixon DP Davies BG Edwards E Functional divergence in the glutathione transferase superfamily in plants J Biol Chem 2002 277 30859 30869 10.1074/jbc.M202919200 12077129

[B14] Loyall L Uchida K Braun S Furuya M Frohnmeyer H Glutathione and a UV light–induced glutathione S-transferase are involved in signaling to chalcone synthase in cell cultures The Plant Cell 2000 12 1939 1950 11041888 10.1105/tpc.12.10.1939 PMC149131

[B15] Kampranis SC Damianova R Atallah M Toby G Kondi G Tsichlis PN Makris AM A novel plant glutathione S-transferase/peroxidase suppresses bax lethality in yeast J. Biol. Chem 2000 275 29207 29216 10.1074/jbc.M002359200 10859306

[B16] Dixon DP Cummins I Cole DJ Edwards R Glutathione-mediated detoxification systems in plants Curr Opin Plant Biol 1998 1 258 266 10.1016/S1369-5266(98)80114-3 10066594

[B17] Urano J Nakagawa T Maki Y Masumura T Tanaka K Murata N Ushimaru T Molecular cloning and characterization of a rice dehydroascorbate reductase FEBS Lett 2000 466 107 111 10.1016/S0014-5793(99)01768-8 10648822

[B18] Jakobsson PJ Morgenstern R Mancini J Ford-Hutchinson A Persson B Common structural features of MAPEG - a widespread superfamily of membrane associated proteins with highly divergent functions in eicosanoid and glutathione metabolism Protein Sci 1999 8 689 692 10091672 10.1110/ps.8.3.689 PMC2144274

[B19] Soranzo N Sari Gorla M Mizzi L De Toma G Frova C Organisation and structural evolution of the rice glutathione S -transferase gene family Mol Gen Genomics 2004 271 511 521 10.1007/s00438-004-1006-8 15069639

[B20] Conn S Curtin C Bézier A Franco C Zhang W Purification, molecular cloning, and characterization of glutathione S-transferases (GSTs) from pigmented *Vitis vinifera* L. cell suspension cultures as putative anthocyanin transport proteins J Exp Bot 2008 13 3621 3634 18836188 10.1093/jxb/ern217 PMC2561157

[B21] Frova C The plant glutathione transferase gene family: genomic structure, functions, expression and evolution Physiologia Plantarum 2003 119 469 479 10.1046/j.1399-3054.2003.00183.x

[B22] Licciardello C Russo MP Valè GP Reforgiato Recupero G Identification of differentially expressed genes in the flesh of blood and common oranges Tree Genetics & Genomes 2008 4 315 331 10.1007/s11295-007-0111-3

[B23] Jain M Ghanashyam C Bhattacharjee A Comprehensive expression analysis suggests overlapping and specific roles of rice glutathione S-transferase genes during development and stress responses BMC Genomics 2010 11 73 90 10.1186/1471-2164-11-73 20109239 PMC2825235

[B24] Sappl PG Carroll AJ Clifton R Lister R Whelan J Harvey Millar A Singh KB The Arabidopsis glutathione transferase gene family displays complex stress regulation and co-silencing multiple genes results in altered metabolic sensitivity to oxidative stress Plant J 2009 58 53 68 10.1111/j.1365-313X.2008.03761.x 19067976

[B25] Lan T Yang ZL Yang X Liu YJ Wang XR Zeng QY Extensive functional diversification of the Populus glutathione S-transferase supergene family Plant Cell 2009 21 3749 66 10.1105/tpc.109.070219 19996377 PMC2814494

[B26] Chi Y Cheng Y Vanitha J Kumar N Ramamoorthy R Ramachandran S Jiang SY Expansion mechanisms and functional divergence of the glutathione S-transferase family in sorghum and other higher plants DNA Research 2011 18 1 16 10.1093/dnares/dsq031 21169340 PMC3041506

[B27] Butelli E Licciardello C Zhang Y Liu J Mackay S Bailey P Reforgiato Recupero G Martin C Retrotransposons control fruit-specific, cold-dependent accumulation of anthocyanins in blood oranges Plant Cell 2012 24 1242 1255 10.1105/tpc.111.095232 22427337 PMC3336134

[B28] Crifò T Petrone G Lo Cicero L Lo Piero AR Short cold storage enhances the anthocyanin contents and level of transcripts related to their biosynthesis in blood oranges J Agric Food Chem 2012 60 476 81 10.1021/jf203891e 22148517

[B29] Sun Y Li H Huang J-R Arabidopsis TT19 functions as a carrier to transport anthocyanin from the cytosol to tonoplasts Molecular Plant 2012 5 387 400 10.1093/mp/ssr110 22201047

[B30] Conn SJ Characterisation of anthocyanin transport and storage in *Vitis vinifera* L. cv. Gamay Fréaux cell suspension cultures PhD thesis 2011 Adelaide: Flinders University

[B31] Winkel BSJ Metabolic channeling in plants Annu 2004 55 85 107 10.1146/annurev.arplant.55.031903.141714 15725058

[B32] Marrs KA Walbot V Expression and RNA splicing of the maize glutathione S-transferase Bronze2 gene is regulated by cadmium and other stresses Plant Physiol 1997 113 93 102 10.1104/pp.113.1.93 9008391 PMC158119

[B33] Smith AP Nourizadeh SD Peer WA Xu J Bandyopadhyay A Murphy AS Goldsbrough PB Arabidopsis AtGSTF2 is regulated by ethylene and auxin, and encodes a glutathione S-transferase that interacts with flavonoids The Plant Journal 2003 36 433 442 10.1046/j.1365-313X.2003.01890.x 14617075

[B34] Mezzari MP Walters K Jelinkova M Shih MC Just CL Schnoor JL Gene expression and microscopic analysis of Arabidopsis exposed to chloroacetanilide herbicides and explosive compounds. A phytoremediation approach Plant Physiol 2005 138 858 869 10.1104/pp.104.056168 15923336 PMC1150403

[B35] Kitamura S Matsuda F Tohge T Yonekura-Sakakibara K Yamazaki M Saito K Narumi I Metabolic profiling and cytological analysis of proanthocyanidins in immature seeds of Arabidopsis thaliana flavonoid accumulation mutants Plant J 2010 62 549 559 10.1111/j.1365-313X.2010.04174.x 20180920

[B36] Kitamura S Shikazono N Tanaka A TRANSPARENT TESTA 19 is involved in the accumulation of both anthocyanins and proanthocyanidins in Arabidopsis Plant J 2004 37 104 114 10.1046/j.1365-313X.2003.01943.x 14675436

[B37] Bernardi J Licciardello C Russo MP Chiusano ML Carletti G Reforgiato Recupero G Marocco A Use of a custom array to study differentially expressed genes during blood orange (*Citrus sinensis* L. Osbeck) ripening J Plant Physiol 2010 167 301 10 10.1016/j.jplph.2009.09.009 19864041

[B38] Saslowsky D Winkel-Shirley B Localization of flavonoid enzymes in *Arabidopsis* roots Plant J 2001 27 37 48 10.1046/j.1365-313x.2001.01073.x 11489181

[B39] Pairoba CF Walbot V Post-transcriptional regulation of expression of the *Bronze2 g*ene of *Zea mays* L Plant Mol Biol 2003 53 75 86 14756308 10.1023/B:PLAN.0000009267.76482.ce

[B40] Lo Piero AR Puglisi I Petrone G Gene isolation, analysis of expression, and *in vitro* synthesis of glutathione s-transferase from orange fruit [*Citrus sinensis* L. (Osbeck)] J Agric Food Chem 2006 54 9227 9233 10.1021/jf0616816 17117814

[B41] Kiyosue T Yamaguchi-Shinozaki K Shinozaki K Characterization of two cDNAs (ERD11 and ERD13) for dehydration-inducible genes that encode putative glutathione S-transferases in *Arabidopsis thaliana* L FEBS Lett 1993 335 189 92 10.1016/0014-5793(93)80727-C 8253194

[B42] Mauch F Dudler R Differential induction of distinct glutathione-S-transferases of wheat by xenobiotics and by pathogen attack Plant Physiol 1993 102 1193 201 10.1104/pp.102.4.1193 8278547 PMC158905

[B43] Zhou J Goldsbrough PB An Arabidopsis gene with homology to glutathione S-transferases is regulated by ethylene Plant Mol Biol 1993 22 517 23 10.1007/BF00015980 8329687

[B44] Chen W Chao G Singh KB The promoter of a H2O2-inducible, Arabidopsis glutathione S-transferase gene contains closely linked OBF- and OBP1-binding sites Plant J 1996 10 955 66 10.1046/j.1365-313X.1996.10060955.x 9011080

[B45] Chen W Singh KB The auxin, hydrogen peroxide and salicylic acid induced expression of the Arabidopsis GST6 promoter is mediated in part by an ocs element Plant J 1999 19 667 77 10.1046/j.1365-313x.1999.00560.x 10571852

[B46] Seppanen MM Cardi T Hyokki MB Pehu E Characterization and expression of cold induced glutathione S-transferase in freezing tolerant *Solanum commersonii*, sensitive *S. tuberosum* and their interspecific somatic hybrids Plant Sci 2000 153 125 33 10.1016/S0168-9452(99)00252-6 10717318

[B47] Vollenweider S Weber H Stolz S Chetelat A Farmer EE Fatty acid ketodienes and fatty acid ketotrienes: Michael addition acceptors that accumulate in wounded and diseased Arabidopsis leaves Plant J 2000 24 467 76 10.1046/j.1365-313x.2000.00897.x 11115128

[B48] Bianchi MW Roux C Vartanian N Drought regulation of GST8, encoding the Arabidopsis homologue of ParC/Nt107 glutathione transferase/peroxidase Physiol. Plant 2002 116 96 105 10.1034/j.1399-3054.2002.1160112.x 12207667

[B49] Moons A Osgstu3 and osgtu4, encoding tau class glutathione S-transferases, are heavy metal- and hypoxic stress-induced and differentially salt stress responsive in rice roots FEBS Lett 2003 553 427 32 10.1016/S0014-5793(03)01077-9 14572664

[B50] Lo Piero AR Mercurio V Puglisi I Petrone G Gene isolation and expression analysis of two distinct sweet orange [*Citrus sinensis* L. (Osbeck)] tau-type glutathione transferases Gene 2009 443 143 150 10.1016/j.gene.2009.04.025 19422890

[B51] Carletti G Lucini L Busconi M Marocco A Bernardi J Insight into the role of anthocyanin biosynthesis-related genes in *Medicago truncatula* mutants impaired in pigmentation in leaves Plant Physiol 2013 70 123 132 10.1016/j.plaphy.2013.05.030 23774374

[B52] Kalyanaraman A Aluru S Kothari S Brendel V Efficient clustering of large EST data sets on parallel computers Nucleic Acids Res 2003 31 2963 2974 10.1093/nar/gkg379 12771222 PMC156714

[B53] Huang X Madan A CAP3: a DNA sequence assembly program Genome Res 1999 9 868 877 10.1101/gr.9.9.868 10508846 PMC310812

[B54] Henikoff S Henikoff JG Embedding strategies for effective use of information from multiple sequence alignments Protein Sci 1997 6 698 705 9070452 10.1002/pro.5560060319 PMC2143675

[B55] Larkin MA Blackshields G Brown NP Chenna R McGettigan PA McWilliam H Valentin F Wallace IM Wilm A Lopez R Thompson JD Gibson TJ Higgins DG ClustalW and ClustalX version 2 Bioinformatics 2007 23 2947 2948 10.1093/bioinformatics/btm404 17846036

[B56] Hall TA BioEdit: a user-friendly biological sequence alignment editor and analysis program for Windows 95/98/NT Nucleic Acids Symposium Series 1999 41 95 98

[B57] Felsenstein J Phylogeny Inference Package (PHYLIP). Version 3.5 1993 Seattle: University of Washington

[B58] Gremme G Brendel V Sparks ME Kurtz S Engineering a software tool for gene structure prediction in higher organisms Information and Software Technology 2005 47 965 978 10.1016/j.infsof.2005.09.005

[B59] Sambrook J Russel DW Cold Spring Harbor Laboratory Molecular cloning: a laboratory manual. Volume 1 2001 3 New York: Plainview

