# Supplementary material for: Characterization of the glutathione S-transferase gene family through ESTs and expression analyses within common and pigmented cultivars of Citrus sinensis (L.) Osbeck
Source: BMC Plant Biol. 2014 Feb 3;14:39. doi: 10.1186/1471-2229-14-39 (PMC3922800; doi:10.1186/1471-2229-14-39)
Supplement: Additional file 1: Figure S1 — Detailed scheme of the procedure used. [file 1471-2229-14-39-S1.pptx]

## Slide 1
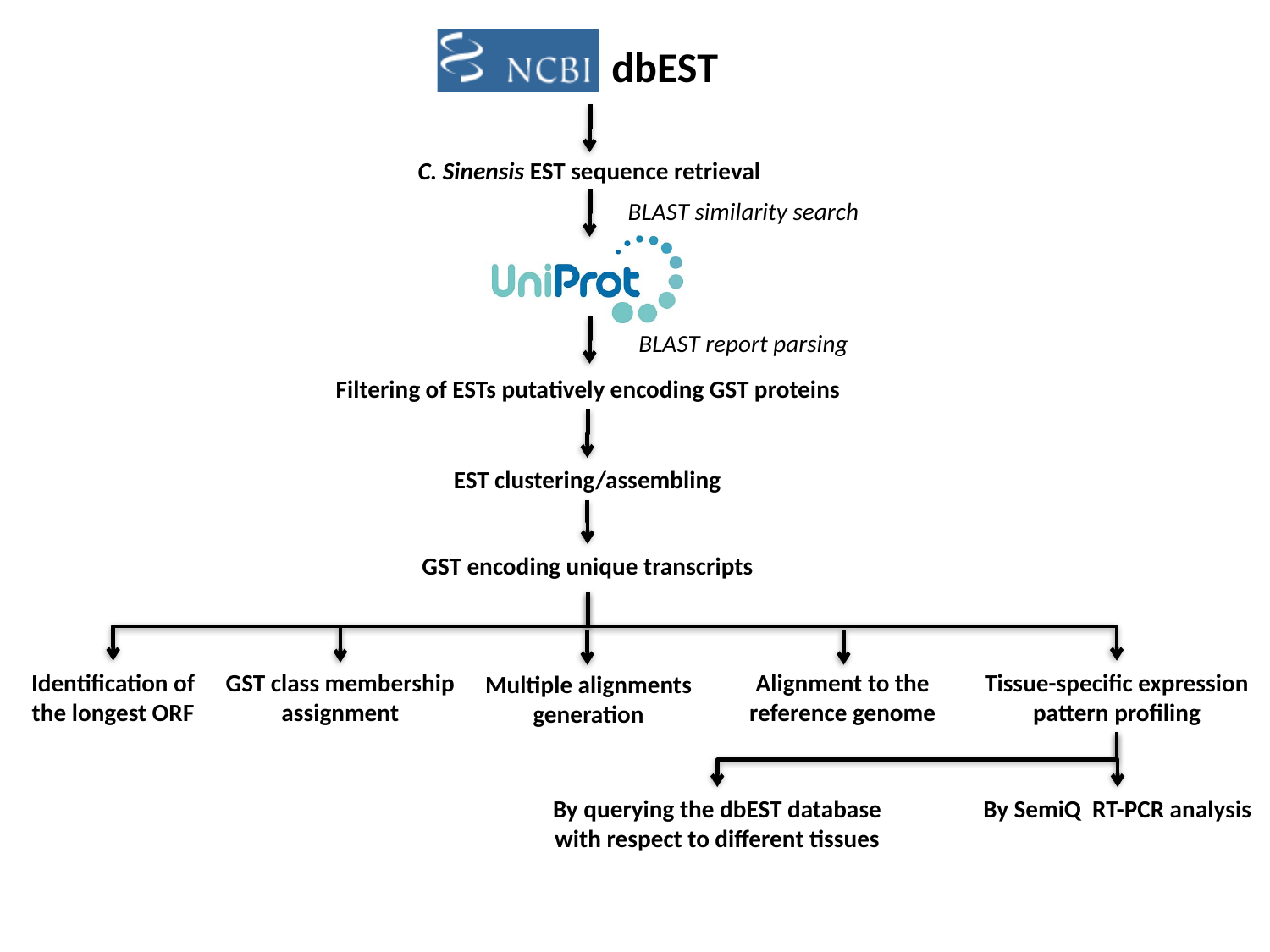

dbEST
C. Sinensis EST sequence retrieval
BLAST similarity search
BLAST report parsing
Filtering of ESTs putatively encoding GST proteins
EST clustering/assembling
GST encoding unique transcripts
Identification of the longest ORF
GST class membership assignment
Alignment to the reference genome
Tissue-specific expression pattern profiling
Multiple alignments generation
By querying the dbEST database with respect to different tissues
By SemiQ RT-PCR analysis
