# Supplementary material for: Characterization of the glutathione S-transferase gene family through ESTs and expression analyses within common and pigmented cultivars of Citrus sinensis (L.) Osbeck
Source: BMC Plant Biol. 2014 Feb 3;14:39. doi: 10.1186/1471-2229-14-39 (PMC3922800; doi:10.1186/1471-2229-14-39)
Supplement: Additional file 3: Table S2 — Schema of GST proteins divided into each class end in relation to dicotyledon (A. thaliana, C. sinensis, V. vinifera, G. max) and monocotyledon (O. sativa, Z. mays) affiliation deducted after protein alignment. The number of encoded proteins for each plant species, the ID of the protein and the genome position are showed; the chromosome start-end for each transcript when possible is also indicated. In the Phi class some examples of clusterization analysis are indicated (a) genes from different chromosomes, (b-c) proximal gene and (d) alternative transcripts. [file 1471-2229-14-39-S3.doc]

| **GST CLASS** | **PLANT CLASS** | **ORGANISM** | **ID** |
| --- | --- | --- | --- |
| **TAU** | Dicotyledon (65) | *A. thaliana (29)* | At|NP_172507.1|chr01|3397083 |
|  |  |  | At\|NP_174033.1\|chr01\|9427151 |
|  |  |  | At\|NP_174034.1\|chr01\|9429034 |
|  |  |  | At\|NP_177150.2\|chr01\|26341160 |
|  |  |  | At\|NP_177151.1\|chr01\|26345464 |
|  |  |  | At\|NP_172508.1\|chr01\|3400607 |
|  |  |  | At\|NP_176176.1\|chr01\|21935498 |
|  |  |  | At\|NP_176178.1\|chr01\|21942032 |
|  |  |  | At\|NP_173160.1\|chr01\|5872138 |
|  |  |  | At\|NP_173161.1\|chr01\|5875343 |
|  |  |  | At\|NP_177955.1\|chr01\|29477785 |
|  |  |  | At\|NP_173162.1\|chr01\|5878405 |
|  |  |  | At\|NP_177956.1\|chr01\|29486963 |
|  |  |  | At\|NP_177957.1\|chr01\|29489162 |
|  |  |  | At\|NP_565178.1\|chr01\|29495788 |
|  |  |  | At\|NP_177958.1\|chr01\|29491306 |
|  |  |  | At\|NP_175772.1\|chr01\|20046308 |
|  |  |  | At\|NP_189966.1\|chr03\|15675358 |
|  |  |  | At\|NP_180503.1\|chr02\|12627137 |
|  |  |  | At\|NP_180505.1\|chr02\|12631663 |
|  |  |  | At\|NP_180506.1\|chr02\|12632931 |
|  |  |  | At\|NP_180507.1\|chr02\|12635618 |
|  |  |  | At\|NP_180508.1\|chr02\|12637275 |
|  |  |  | At\|NP_180509.1\|chr02\|12638455 |
|  |  |  | At\|NP_180510.1\|chr02\|12640168 |
|  |  |  | At\|NP_187538.1\|chr03\|2850926 |
|  |  |  | At\|NP_851249.1\|chr05\|25105920 |
|  |  |  | At\|NP_568954.2\|chr05\|25107356 |
|  |  |  | At\|NP_177598.1\|chr01\|28028814 |
|  |  | *C. sinensis (12)* | Cs\|CITSI47_3 |
|  |  |  | Cs\|CITSI57:1:1_1 |
|  |  |  | Cs\|CITSI43:1:1_1 |
|  |  |  | Cs\|CITSI16:1_1 |
|  |  |  | Cs\|CITSI51:1_1 |
|  |  |  | Cs\|CITSI36:1_1 |
|  |  |  | Cs\|CITSI44:1_1 |
|  |  |  | Cs\|CITSI24_CX077363 |
|  |  |  | Cs\|CITSI41:1_1 |
|  |  |  | Cs\|CITSI04:1_1 |
|  |  |  | Cs\|CITSI53:1_1 |
|  |  |  | Cs\|CITSI25:1_1 |
|  |  | *V. vinifera (3)* | Vv(GSVIVP00006652001) |
|  |  |  | Vv(GSVIVP00006659001) |
|  |  |  | Vv(GSVIVP00022906001) |
|  |  | *G. max (21)* | Gm\|AAG34795.1 |
|  |  |  | Gm\|AAC18566.1 |
|  |  |  | Gm\|AAG34800.1 |
|  |  |  | Gm\|CAA71784.1 |
|  |  |  | Gm\|CAA48717.1 |
|  |  |  | Gm\|AAG34796.1 |
|  |  |  | Gm\|AAG34801.1 |
|  |  |  | Gm\|AAG34802.1 |
|  |  |  | Gm\|AAG34803.1 |
|  |  |  | Gm\|AAG34810.1 |
|  |  |  | Gm\|AAG34804.1 |
|  |  |  | Gm\|AAG34805.1 |
|  |  |  | Gm\|AAG34806.1 |
|  |  |  | Gm\|AAA33943.1 |
|  |  |  | Gm\|AAA33973.1 |
|  |  |  | Gm\|AAG34797.1 |
|  |  |  | Gm\|AAG34798.1 |
|  |  |  | Gm\|AAG34799.1 |
|  |  |  | Gm\|AAG34807.1 |
|  |  |  | Gm\|AAG34808.1 |
|  |  |  | Gm\|AAG34809.1 |
|  | Monocotyledon (86) | *O. sativa (58)* | Os\|NP_917039.1\|chr01\|28894471 |
|  |  |  | Os\|NP_917040.1\|chr01\|28896613 |
|  |  |  | Os+\|Os07g07320\|chr07\|3660710 3 |
|  |  |  | Os+\|Os01g37750\|chr01\|21442537 |
|  |  |  | Os\|NP_921049.1\|chr10\|11066947 |
|  |  |  | Os\|NP_922426.1\|chr10\|61.7cM |
|  |  |  | Os\|NP_922427.1\|chr10\|20108236 |
|  |  |  | Os\|NP_922442.1\|chr10\|20159106 |
|  |  |  | Os\|NP_922443.1\|chr10\|20163743 |
|  |  |  | Os\|AAK98540.1\|chr10\|20168407 |
|  |  |  | Os\|NP_922455.1\|chr10\|20221601 |
|  |  |  | Os\|NP_922456.1\|chr10\|20224024 |
|  |  |  | Os\|NP_922468.1\|chr10\|20265421 |
|  |  |  | Os\|AAS86424.1\|chr10\|61.7cM |
|  |  |  | Os\|AAG32470.1\|chr09\|12120224 |
|  |  |  | Os+\|Os10g34020\|chr10\|17820048 |
|  |  |  | Os\|AAG32471.1\|chr10\|20226791 |
|  |  |  | Os\|NP_922472.1\|chr10\|20274076 |
|  |  |  | Os\|NP_922474.1\|chr10\|20277801 |
|  |  |  | Os\|AAK98535.1\|chr10\|61.7cM |
|  |  |  | Os\|AAK98536.1\|chr10\|20267457 |
|  |  |  | Os\|NP_922475.1\|chr10\|20280491 |
|  |  |  | Os\|AAG32472.1\|chr10\|20229972 |
|  |  |  | Os\|AAK98541.1\|chr10\|20293774 |
|  |  |  | Os\|AAK98537.1\|chr10\|20262960 |
|  |  |  | Os\|AAK98542.1\|chr10\|20245238 |
|  |  |  | Os\|NP_922477.1\|chr10\|20287129 |
|  |  |  | Os\|AAK98543.1\|chr10\|20103431 |
|  |  |  | Os+\|Os10g38150\|chr10\|20105899 |
|  |  |  | Os+\|Os10g38189\|chr10\|20120356 |
|  |  |  | Os\|AAK98538.1\|chr10\|61.7cM |
|  |  |  | Os+\|Os10g38630\|chr10\|20271291 |
|  |  |  | Os\|AAS86425.1\|chr10\|11206308 |
|  |  |  | Os\|AAK98544.1\|chr5\|77.4cM |
|  |  |  | Os+\|Os10g38720\|chr10\|20299448 |
|  |  |  | Os\|NP_922478.1\|chr10\|20290739 |
|  |  |  | Os+\|Os10g38730\|chr10\|20301464 |
|  |  |  | Os+\|Os10g38740\|chr10\|20304286 |
|  |  |  | Os+\|Os10g38780\|chr10\|20316311 |
|  |  |  | Os\|NP_921310.1\|chr10\|12934134 |
|  |  |  | Os+\|Os07g28480\|chr07\|16672702 |
|  |  |  | Os+\|Os03g57200\|chr03\|32559090 |
|  |  |  | Os\|AAC05216.1\|chr3\|146.1cM |
|  |  |  | Os\|AAG32473.1\|chr10\|61.7cM |
|  |  |  | Os+\|Os03g44170\|chr03\|24780687 |
|  |  |  | Os+\|Os11g03210\|chr11\|1165520 1 |
|  |  |  | Os+\|Os12g02960\|chr12\|1096623 1 |
|  |  |  | Os+\|Os01g72120\|chr01\|42166847 |
|  |  |  | Os+\|Os03g39850\|chr03\|22116101 |
|  |  |  | Os+\|Os01g72130\|chr01\|42168609 |
|  |  |  | Os+\|Os01g72140\|chr01\|42170907 |
|  |  |  | Os+\|Os01g72150\|chr01\|42173295 |
|  |  |  | Os+\|Os01g72160\|chr01\|42174579 |
|  |  |  | Os+\|Os01g72170\|chr01\|42180906 |
|  |  |  | Os\|AAK98545.1\|chr09\|17746300 |
|  |  |  | Os+\|Os05g34150\|chr05\|20110504 |
|  |  |  | Os\|AAK98546.1\|chr10\|20260145 |
|  |  |  | Os\|AAK98539.1\|chr7\|26 31cM |
|  |  | *Z. mays (28)* | Zm\|AAG34827.1 |
|  |  |  | Zm\|AAG34830.1 |
|  |  |  | Zm\|AAG34840.1 |
|  |  |  | Zm\|AAG34831.1 |
|  |  |  | Zm\|AAG34850.1 |
|  |  |  | Zm\|AAG34841.1 |
|  |  |  | Zm\|AAG34842.1 |
|  |  |  | Zm\|AAG34843.1 |
|  |  |  | Zm\|AAG34844.1 |
|  |  |  | Zm\|AAG34845.1 |
|  |  |  | Zm\|AAG34832.2 |
|  |  |  | Zm\|AAG34846.1 |
|  |  |  | Zm\|AAG34833.1 |
|  |  |  | Zm\|AAG34834.1 |
|  |  |  | Zm\|AAG34847.1 |
|  |  |  | Zm\|AAG34835.1 |
|  |  |  | Zm\|AAA50245.1 |
|  |  |  | Zm\|AAG34828.1 |
|  |  |  | Zm\|CAA73369.1 |
|  |  |  | Zm\|CAB38120.1 |
|  |  |  | Zm\|CAB38121.1 |
|  |  |  | Zm\|AAG34836.1 |
|  |  |  | Zm\|AAG34829.1 |
|  |  |  | Zm\|AAG34837.1 |
|  |  |  | Zm\|AAG34838.1 |
|  |  |  | Zm\|AAG34848.1 |
|  |  |  | Zm\|AAG34839.1 |
|  |  |  | Zm\|AAG34849.1 |
| **PHI** | Dicotyledon (33) | *A. thaliana (18)* | At|NP_178394.1|chr02|854946^a^ |
|  |  |  | At\|NP_192161.1\|chr04\|1112141^a^ |
|  |  |  | At\|NP_171791.1\|chr01\|661177^b^ |
|  |  |  | At\|NP_171792.1\|chr01\|663079 ^b^ |
|  |  |  | At\|NP_850479.1\|chr02\|19567197 |
|  |  |  | At\|NP_171793.1\|chr01\|665152^d^ |
|  |  |  | At\|NP_849581.1\|chr01\|665152 ^d^ |
|  |  |  | At\|NP_563670.1\|chr01\|665152 ^d^ |
|  |  |  | At\|NP_001030937.1\|chr01\|665152^d^ |
|  |  |  | At\|NP_180643.1\|chr02\|13148434 |
|  |  |  | At\|NP_180644.1\|chr02\|13149742 |
|  |  |  | At\|NP_001077983.1\|chr02\|131460 |
|  |  |  | At\|NP_186969.1\|chr03\|736148 |
|  |  |  | At\|NP_197224.1\|chr05\|5659576 |
|  |  |  | At\|NP_191835.1\|chr03\|23229609 |
|  |  |  | At\|NP_175408.1\|chr01\|18463880^a^ |
|  |  |  | At\|NP_177853.1\|chr01\|29043269^c^ |
|  |  |  | At\|NP_001031292.1\|chr01\|290448^c^ |
|  |  | *C. sinensis (10)* | Cs\|CITSI52:1_1 |
|  |  |  | Cs\|CITSI23:1_1 |
|  |  |  | Cs\|CITSI33:1_1 |
|  |  |  | Cs\|CITSI05:DY257328_DY257328 |
|  |  |  | Cs\|CITSI34:CK939385_CK939385 |
|  |  |  | Cs\|CITSI38:CK934228_CK934228 |
|  |  |  | Cs\|CITSI11:1_1 |
|  |  |  | Cs\|DQ198153\|CITSI02 CITSI00 |
|  |  |  | Cs\|CITSI18:1_1 |
|  |  |  | Cs\|CITSI29:1_1 |
|  |  | *V. vinifera (2)* | Vv(GSVIVP00018860001) |
|  |  |  | Vv(GSVIVP00027954001) |
|  |  | *G. max (3)* | Gm\|AAG34812.1 |
|  |  |  | Gm\|AAG34814.1 |
|  |  |  | Gm\|AAG34811.1 |
|  | Monocotyledon (32) | *O. sativa (20)* | Os\|AAG32475.1\|chr01\|15498666 |
|  |  |  | Os+\|Os01g25100\|chr01\|14149730 |
|  |  |  | Os\|CAA05354.1\|chr01\|15609819 |
|  |  |  | Os\|NP_918725.1\|chr01\|15587092 |
|  |  |  | Os\|NP_918731.1\|chr01\|15618603 |
|  |  |  | Os\|NP_918749.1\|chr01\|15737704 |
|  |  |  | Os\|AAC64007.1\|chr01\|32478700 |
|  |  |  | Os+\|Os01g27300\|chr01\|15561311 |
|  |  |  | Os\|NP_918719.1\|chr01\|15544818 |
|  |  |  | Os\|NP_9187380.1\|chr01\|15616080 |
|  |  |  | Os+\|Os05g05620\|chr05\|2774511 |
|  |  |  | Os+\|Os01g27480\|chr01\|15671332 |
|  |  |  | Os\|AAG32476.1\|chr01\|41290933 |
|  |  |  | Os+\|Os03g04250\|chr03\|1932502 |
|  |  |  | Os\|AAS86423.1\|chr03\|1928546 |
|  |  |  | Os\|AAS86442.1\|chr03\|1918711 |
|  |  |  | Os\|NP_922584.1\|chr10\|20903311 |
|  |  |  | Os\|AAG32477.1\|chr03\|1934992 |
|  |  |  | Os+\|Os09g37240\|chr09\|21496134 |
|  |  |  | Os+\|Os08g43680\|chr08\|27476503 |
|  |  | *Z. mays (12)* | Zm\|AAG34816.1 |
|  |  |  | Zm\|CAA56047.1 |
|  |  |  | Zm\|AAG34823.1 |
|  |  |  | Zm\|AAA33470.1 |
|  |  |  | Zm\|CAA28053.1 |
|  |  |  | Zm\|AAG34817.1 |
|  |  |  | Zm\|AAG34820.1 |
|  |  |  | Zm\|AAG34821.1 |
|  |  |  | ZmAAG34822.1 |
|  |  |  | Zm\|AAG34822.1 |
|  |  |  | Zm\|AAG34818.1 |
|  |  |  | Zm\|AAG34824.1 |
| **LAMBDA** | Dicotyledon (6) | *A. thaliana (4)* | At|NP_191064.1|chr03|20411465 |
|  |  |  | At\|NP_568336.1\|chr05\|5485356 5 |
|  |  |  | At\|NP_195898.2\|chr05\|630874 63 |
|  |  |  | At\|NP_195899.1\|chr05\|635226 63 |
|  |  | *C. sinensis (1)* | CITSI46:1_1 |
|  |  | *V. vinifera (1)* | Vv(XP_002275882) |
| **ZETA** | Dicotyledon (7) | *A. thaliana (4)* | At|NP_178344.1|chr02|628898 63 |
|  |  |  | At\|NP_973400.1\|chr02\|628898 63 |
|  |  |  | At\|NP_849926.2\|chr02\|631235 63 |
|  |  |  | At\|NP_178343.1\|chr02\|628898 63 |
|  |  | *C. sinensis (1)* | Cs\|CITSI21:1_1 |
|  |  | *V. vinifera (1)* | Vv(XP_002273077) |
|  |  | *G. max (1)* | Gm\|AAG34815.1 |
|  | Monocotyledon (7) | *O. sativa (5)* | Os+\|Os12g10730\|chr12\|5759624 5 |
|  |  |  | Os\|AAG32474.1\|chr12\|5755506â€“ |
|  |  |  | Os\|AAS83978.1\|chr02\|21407729 2 |
|  |  |  | Os\|AAK98533.1\|chr12\|39.7cM |
|  |  |  | Os+\|Os11g14040\|chr11\|7787757 7 |
|  |  | *Z. mays (2)* | Zm\|AAG34826.1 |
|  |  |  | Zm\|AAG34825.1 |
| **MAPEG** | Dicotyledon (2) | *A. thaliana (1)* | At|NP_176758.1|chr01|24493443 |
|  |  | *C. sinensis (1)* | CITSI20:1_1 |
|  | Monocotyledon (1) | *O. sativa (1)* | Os+\|Os03g50130\|chr03\|28545404 |
| **THETA** | Dicotyledon (5) | *A. thaliana (3)* | At|NP_198938.1|chr05|16515383 |
|  |  |  | At\|NP_198940.3\|chr05\|16518445 |
|  |  |  | At\|NP_198937.1\|chr05\|16511654 |
|  |  | *V. vinifera (1)* | vv(GSVIVP00000406001) |
|  |  | *G. max (1)* | Gm\|AAG34813.1 |
|  | Monocotyledon (1) | *O. sativa (1)* | Os\|AKK98534.1\|chr11\|21804678 |
| **DHAR** | Dicotyledon (5) | *A. thaliana (4)* | At|NP_177662.1|chr01|28255621 |
|  |  |  | At\|NP_173387.1\|chr01\|6773293 6 |
|  |  |  | At\|NP_001077564.1\|chr01\|677475 |
|  |  |  | At\|NP_173386.1\|chr01\|6773293 6 |
|  |  | *V. vinifera (1)* | Vv(XP_002282399.1) |
